# Smart Industrial Internet of Things Framework for Composites Manufacturing

**DOI:** 10.3390/s24154852

**Published:** 2024-07-26

**Authors:** Boon Xian Chai, Maheshi Gunaratne, Mohammad Ravandi, Jinze Wang, Tharun Dharmawickrema, Adriano Di Pietro, Jiong Jin, Dimitrios Georgakopoulos

**Affiliations:** 1 Aerostructures Innovation Research Hub (AIR Hub), Swinburne University of Technology, Hawthorn, VIC 3122, Australia; bchai@swin.edu.au (B.X.C.);; 2 School of Science, Computing and Engineering Technologies, Swinburne University of Technology, Hawthorn, VIC 3122, Australia; 3 ARC Industrial Transformation Research Hub for Future Digital Manufacturing, Swinburne University of Technology, Hawthorn, VIC 3122, Australia

**Keywords:** Digital Manufacturing, Composites manufacturing, Process simulation, Industrial Internet of Things (IIoT), Artificial Intelligence (AI)

## Abstract

Composite materials are increasingly important in making high-performance products. However, contemporary composites manufacturing processes still encounter significant challenges that range from inherent material stochasticity to manufacturing process variabilities. This paper proposes a novel smart Industrial Internet of Things framework, which is also referred to as an Artificial Intelligence of Things (AIoT) framework for composites manufacturing. This framework improves production performance through real-time process monitoring and AI-based forecasting. It comprises three main components: (i) an array of temperature, heat flux, dielectric, and flow sensors for data acquisition from production machines and products being made, (ii) an IoT-based platform for instantaneous sensor data integration and visualisation, and (iii) an AI-based model for production process forecasting. Via these components, the framework performs real-time production process monitoring, visualisation, and prediction of future process states. This paper also presents a proof-of-concept implementation of the framework and a real-world composites manufacturing case study that showcases its benefits.

## 1. Introduction

The Internet of Things (IoT) refers to the interconnected network of physical devices, vehicles, home appliances, and other items embedded with electronics, software, sensors, actuators, and connectivity, which enables them to connect and exchange data. These devices collect and transmit data over the internet without human intervention, creating opportunities for automation, efficiency improvements, and new services. Ongoing research in Industrial IoT (IIoT) extends IoT capability to enable the creation of real-time-updated representations of complex physical entities, such as industrial machines and products, which are manifested as digital twins (e.g., digital models). Artificial Intelligence of Things (AIoT), in turn, represents the seamless integration of artificial intelligence (AI) technologies into the IoT and IIoT ecosystems [1,2,3]. While IoT platforms integrate process sensors [4,5] to observe physical entities in the real world and to measure their properties [6,7], emerging AIoT technology extends contemporary IoT and IIoT functionality to enable prediction of production outcomes and intelligent decision-making to manage production in both open- and closed-loop settings [3,6,8]. More specifically, AI models integrated into IoT and IIoT solutions could learn from sensor data, machine settings, and parameters, and then predict production outcomes. These predictions can be used to streamline production processes and continuously improve their performance with minimal human intervention. This transformative synergy between AI and IIoT is poised to revolutionise industries by unlocking new levels of efficiency, productivity, and innovation across various sectors.

### State-of-the-Art in Composites Manufacturing

**Figure 1:**
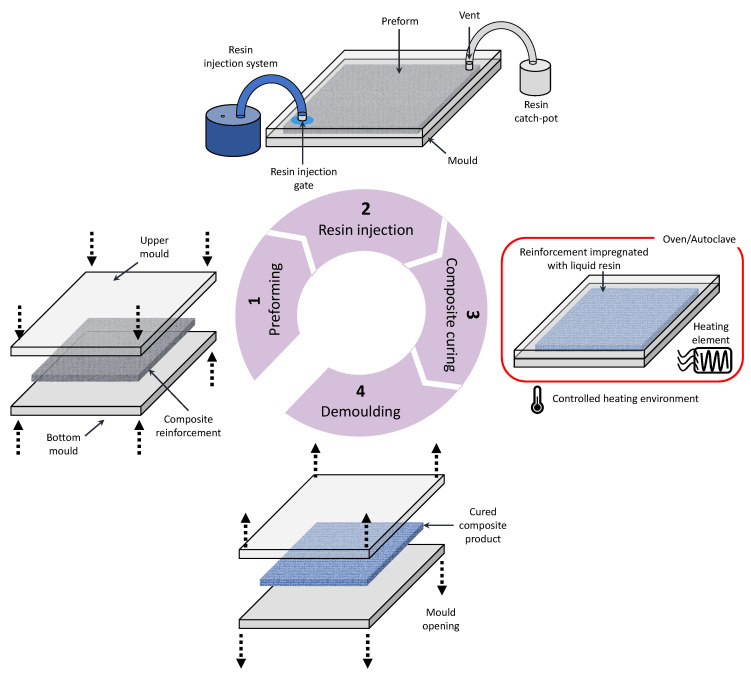
Typical manufacturing stages of the LCM processes.

Recent advancements in AIoT and IIoT, which in the rest of this paper we simply refer to as AIoT, hold great potential to revolutionise the traditional composites manufacturing industry. Composites, especially carbon fibre reinforced composites (CFRP), are frequently adopted across various critical industries such as aerospace, automotive, and defence due to their lightweight and superior specific strength [9,10,11]. The liquid composite moulding (LCM) process family is an out-of-autoclave composites manufacturing method widely used in the composites manufacturing industry. Ranging from the basic resin infusion (RI) to the more sophisticated resin transfer moulding (RTM), LCM processes generally incorporate the distinct manufacturing stages of (1) preforming, (2) resin injection, (3) composite curing, and (4) demoulding [9,12,13]. These LCM stages are depicted in Figure 1. The preforming stage involves shaping the reinforcement fabric into a near-net shape, often with the aid of pressure and heat. In the resin injection stage (which is also known as the mould filling stage), the shaped preform will be injected and wetted with resins inside a mould. The impregnated reinforcement will then be left inside the mould to cure, often aided by pressure or heating as well. Upon complete curing, the composite product can be demoulded.

Contemporary composites manufacturing practices are relatively rudimentary in terms of digitalisation and technological adoptions as compared to other manufacturing sectors [14,15]. Research into improving the productivity and robustness of LCM processes via AI-driven IIoT digital manufacturing techniques is rapidly growing in academic literature, but industry adoption has yet to occur at a similar scale [14,16,17,18]. In the literature, AI technologies have been implemented to model material digital twins [19,20] and process digital twins (composite cure [21], mould filling [11], and demoulding and inspection [22]). Concurrently, IoT capabilities, assisted by AI, have been applied to model and streamline the automatic tape laying (ATP) process [15] and the creation of digital twins for life-cycle and cost assessments for composites [18]. The lack of these applications in industrial settings reflects the slow transition of contemporary composites manufacturing to Industry 4.0 or the more recently debated concept, Composites 4.0 [14].

## 2. AIoT Framework for Composites Manufacturing

One of the main challenges faced by the composites manufacturing industry is the low yield rate [23,24]. On top of lengthy manufacturing processes, the said industry faces two key challenges: material variabilities and process inconsistencies [10,14,15,21]. Variability in material properties is a widespread issue in the industry due to the nature of the raw materials itself, which is an uncontrollable factor. Such material variabilities apply to both the resin and the reinforcing fibres that constitute the composite product. For instance, the high-temperature manufacture of reinforcing fibres often results in the formation of micropores on the fibres, causing unpredictable changes in the fibres’ performance [25,26]. Conversely, process inconsistencies in composites manufacturing can be minimised significantly, albeit often at a high cost [25,27]. Some common process variabilities in composites manufacturing include uneven resin distribution and cure rates, non-uniform clamping and heating rates, and, most commonly, the occurrence of race-tracking, where inconsistencies in reinforcement placements within the mould create unaccounted gaps [21,25,27,28]. To address the aforementioned challenges, contemporary industrial practices fall into two main strategies. The first strategy involves allocating a large ‘factor of safety’ in both materials and process designs to overcompensate for the uncertainties in composites manufacturing. Often practised in critical sectors such as aerospace, the overcommitment of resources results in a non-optimised process, negatively impacting manufacturing efficiency, cost, or both [3,13,14]. Alternatively, the second strategy relies on extensive manual inspection and rework (or scrapping) to ensure product quality instead of resource overcommitment, although such a process is known to potentially take up to 42% of the total time per part manufactured [15].

It is apparent that the traditional LCM processes can be greatly advanced and streamlined through the introduction of AIoT technologies [7,14,15]. In fact, the development of autonomous composites manufacturing systems has been widely discussed in recent years [11,14,15]. In particular, the need for in situ composite defect detection has greatly promoted the development of specialised sensors in the market (e.g., Synthesites [29]). If integrated with IoT capabilities, these specialised sensors can be introduced into any IoT platform, which can unify, collect, and integrate measurements from multiple sensors. It is observed that the individual components of a potentially autonomous Industry 4.0 composites manufacturing system already exist [8,11,29]. The next step forward would be to amalgamate them into a closed-loop system.

To address the contemporary composites manufacturing challenges, in this paper we propose a novel AIoT framework for composites manufacturing. The AIoT framework aims to streamline the composites manufacturing process via real-time process monitoring, which enables the identification of process deviations and allows real-time adjustments or corrections to be performed based on sensed conditions (manually or automatically). The AIoT framework comprises three main components: (i) an array of specialised sensors for process data acquisition, (ii) an IoT-based platform for instantaneous sensor data integration and visualisation, and (iii) an AI-based model for real-time-updated process forecasting. Via these data collection, integration, and analysis capabilities, this novel AIoT framework enables real-time process monitoring, visualisation, and prediction of future process states.

A proof-of-concept implementation of the novel AIoT framework for composite manufacturing was developed using commercially available temperature, dielectric, heat flux, and resin flow sensors alongside commercial IoT platforms and the development of an AI model for process forecasting. The proof-of-concept implementation developed in this study focuses on the resin injection and curing stages as they largely determine the resultant manufacturing efficiency and final product quality [30,31,32]. It is of great interest to monitor and forecast these stages, more specifically, the thermal profile of the mould and the composite while the part is being made, the resin flow progression, the heat transfer behaviour, and the evolution of resin cure [19,33,34].

### Proof-of-Concept Implementation

**Figure 2:**
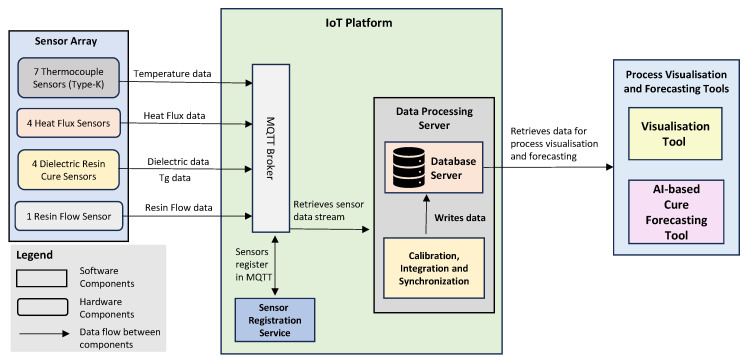
Architecture of AIoT framework for composites manufacturing.

Figure 2 depicts the architecture of the novel AIoT framework for composites manufacturing that reduces the curing cycle while ensuring production quality and efficiency. As depicted in Figure 2, the AIoT framework for composites manufacturing consists of three main components:

- A sensor array of temperature, heat flux, dielectric, and flow sensors for data acquisition from production machines and products being made;
- An IoT platform for sensor data acquisition (DAQ), synchronisation, integration, and management;
- A real-time resin cure monitoring and visualisation tool;
- An AI-based resin cure forecasting tool.

This novel AIoT framework enables data acquisition, integration, synchronisation, and storage; enables data visualisation during manufacture; and supports data-driven process forecasting. In essence, process data from the sensors are fed into the IoT platform, which acquires and synchronises the data collected. The data are then published onto a cloud server, enabling the use of process visualisation and forecasting tools. If deviations from the desired process progression are detected or forecasted, the AIoT framework will automatically adjust the process parameters to compensate for the undesirable deviations.

The sensor array within the proof-of-concept implementation comprises specialised sensors designed to monitor critical aspects of the resin injection and composite curing stages during composites manufacturing. These critical process parameters fall into two main categories: (i) thermal properties and (ii) resin properties. The acquisition of the thermal profile and resin cure evolution data streams currently represents the most reliable data source for monitoring and forecasting the LCM resin injection and curing stages [14,21,35].

It is critical to monitor the composite’s temperature profile during curing, especially when using resins that exhibit exothermic curing behaviour, to prevent exothermic runaway or thermal degradation [21,35,36]. By continuously monitoring the resin, composite, and oven environment temperatures, the heat transfer phenomenon can be reliably captured. These data are also essential for establishing boundary conditions for subsequent process modelling and forecasting. In addition to temperature, it is also important to capture the rate of heat transfer (i.e., convection, conduction, and radiation) during the curing process. While various temperature and heat flux sensor options are available, sensor selection depends on specific application requirements such as accuracy, response time, and size. For the proof-of-concept implementation, thermocouples (Type-K [37]) and heat flux sensors (Hukseflux FHF06 [38]) were chosen for their great accuracy and short response times.

To accurately monitor the mould filling and resin curing progression, it is also essential to track the resin flow and resin dielectric properties. This entails the employment of flow sensors, which detect the resin flow progression within the mould, and dielectric sensors, which monitor changes in resin resistivity to determine the curing status of the resin. These data provide insight into the resin’s glass transition temperature (Tg), which is a reliable indicator of the final cured composite’s mechanical properties. Both the resin flow and resin dielectric sensors adopted in the proof-of-concept implementation were procured from Synthesites [29], an established leader in the field of composites manufacturing and testing. During the proof-of-concept implementation, each sensor type is strategically positioned and programmed to sample data at frequencies optimised for their specific tasks: every second for heat flux sensors and flow sensors, every ten seconds for thermocouples, and every three minutes for dielectric sensors. This meticulous setup ensures precision and consistency in monitoring and data collection. All deployed sensors are integrated with IoT capabilities to enable real-time monitoring of the resin injection and composite curing stages.

The IoT platform is at the core of the AIoT framework, and it consists of (1) a message queuing telemetry transport (MQTT) [39] broker for acquiring the sensor data from the sensor array and (2) a data processing server that calibrates, synchronises, and integrates the sensor data streams before they are stored in its database. In our proof-of-concept implementation, data transfer from sensors to the IoT platform employs the MQTT protocol, which uses a publish–subscribe mechanism. The sensors collect process data and publish them to designated MQTT broker topics at their respective intervals. MQTT was chosen for its scalability, reliability, and secure communication capabilities. It supports various levels of quality of service (QoS), ensuring reliable message delivery even with network issues, and provides secure communication channels through TLS/SSL encryption. This makes MQTT highly suitable for accommodating an increasing number of sensors and data streams without requiring changes to the IIoT architecture. The IIoT platform can use any available MQTT broker implementation, including open-source options or those from commercial IoT platforms like Azure IoT and Amazon AWS IoT.

As noted above, the IoT platform’s data processing server receives the sensor data streams via MQTT and performs data calibration, synchronisation, and integration. Data synchronisation ensures uniformity in data timestamps and deals with the different time intervals of data that arrive from the heat flux sensors, thermocouples, and dielectric sensors. Data integration performs sensor data translation to information at the process level. This functionality of the IoT platform’s data processing server prepares the data monitoring, visualisation, and analysis. In the proof-of-concept implementation of the IoT platform’s data processing server, the calibration, synchronisation, and integration functionality is implemented in Python Scripts, and the Microsoft Azure Database for PostgreSQL [40] is used for data storage. Microsoft Azure is capable of managing large volumes of data collected from the process sensors, offers extensive computational resources, and provides a platform for the integration of AI to predict curing time and product quality.

This cloud-based implementation takes advantage of scalable technologies to accommodate the demanding needs of composite manufacturing processes. The resin cure monitoring and visualisation tool provides real-time monitoring and visualisation of the resin injection and composite curing stages of the LCM process. The proof-of-concept implementation of these tools uses Power BI [41] to provide visualisation of the manufacturing process and offer insights that help in closed- or open-loop decision-making.

The AI-based resin cure forecasting tool used a predictive AI model that can predict the curing time and related product quality/consistency. To develop the AI model, we used data collected from previously conducted resin infusion experiments. As it is both expensive and time-consuming to collect the required volume of training data, we also generated additional data using finite element modelling. During a resin infusion run, every second, the AI model connects to the IoT server database to receive the ten previous sensor readings. The fully trained and validated model will use these readings to predict the next ten sensor readings. These predictions are sent to the visualisation tool (which was implemented using Power BI) to generate a real-time visualisation of the previous readings and predicted trends in one visualisation.

**Figure 3:**
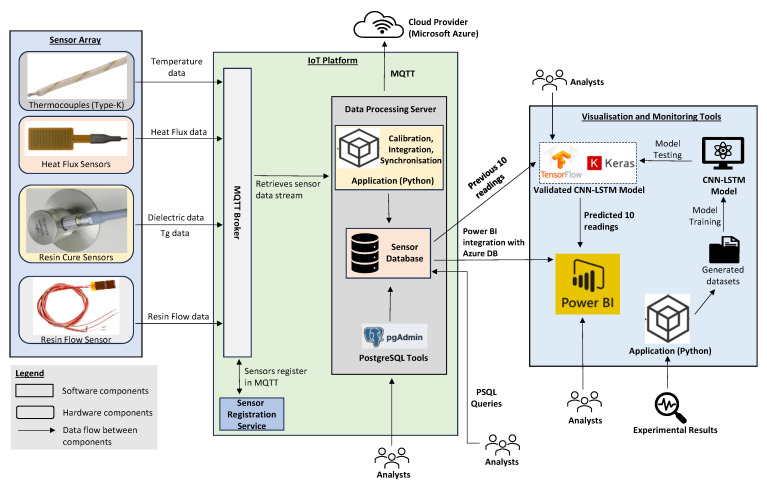
Proof-of-concept implementation of the AIoT framework for composites manufacturing.

The AIoT framework aims to provide robust and secure communication across all components. In the proof-of-concept implementation, the sensors and computing components are connected with Ethernet cables. While this provides a stable and fast data link essential for close-to-real-time data transfer, Ethernet can be easily substituted with fully compatible alternatives, such as ProfiNet, for enhanced reliability. ProfiNet, an industrial Ethernet standard, offers deterministic performance and robustness in industrial environments. The proof-of-concept implementation of the AIoT framework for composite manufacturing is illustrated in Figure 3.

The proof-of-concept implementation of the AIoT framework for composite manufacturing demonstrates how the integrated use of MQTT, data processing, artificial intelligence, and robust communication protocols can enhance the efficiency and reliability of the LCM processes. To verify robustness and security, reliability tests were conducted by intentionally introducing network disruptions such as latency, packet loss, and forced disconnections to the proof-of-concept application. These tests ensured that the system maintained message delivery and integrity. Security testing, including penetration tests, verified the effectiveness of TLS/SSL encryption, preventing unauthorised access and ensuring data confidentiality and integrity. These tests confirmed the robustness and security of the communication framework.

## 3. Case Study: Utilising the Proof-of-Concept Implementation for the Resin Infusion Process

**Figure 4:**
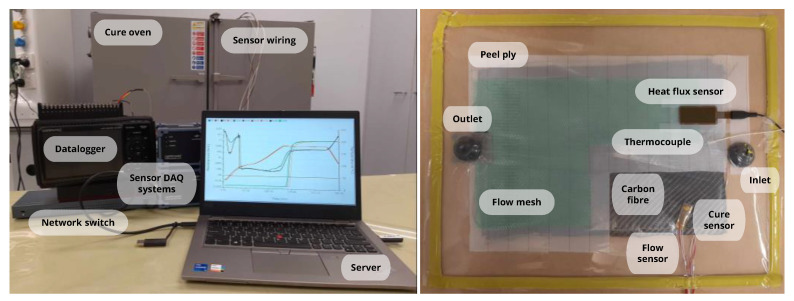
Hardware setup of the AIoT framework (**left**) and the mounting of various sensors and process consumables (peel ply, flow mesh, etc.) on the composite infusion layup (**right**).

This section presents a case study involving an in-house resin infusion (RI) process that (1) utilises the proof-of-concept implementation of the proposed AIoT framework that consists of both hardware and software and (2) illustrates the benefits of the framework. RI is a low-cost composites manufacturing process frequently practised in the industry due to its ability to manufacture large composite products. For the RI process, visual monitoring of process progression poses challenges due to the utilisation of process consumables such as the peel ply, flow mesh, inlets, and outlets. This necessitates the use of alternative sensory techniques. The hardware setup of the proof-of-concept implementation is depicted in Figure 4 (left). The RI process begins with laying up the reinforcement (carbon fibre in this study) and process consumables, including peel ply, flow media, and inlet and outlet gates. Various sensors are then mounted onto the infusion layup, as illustrated in Figure 4 (right). After preparing the composite infusion layup, the entire setup is placed into a composite curing oven for the resin injection and composite curing stages.

**Figure 5:**
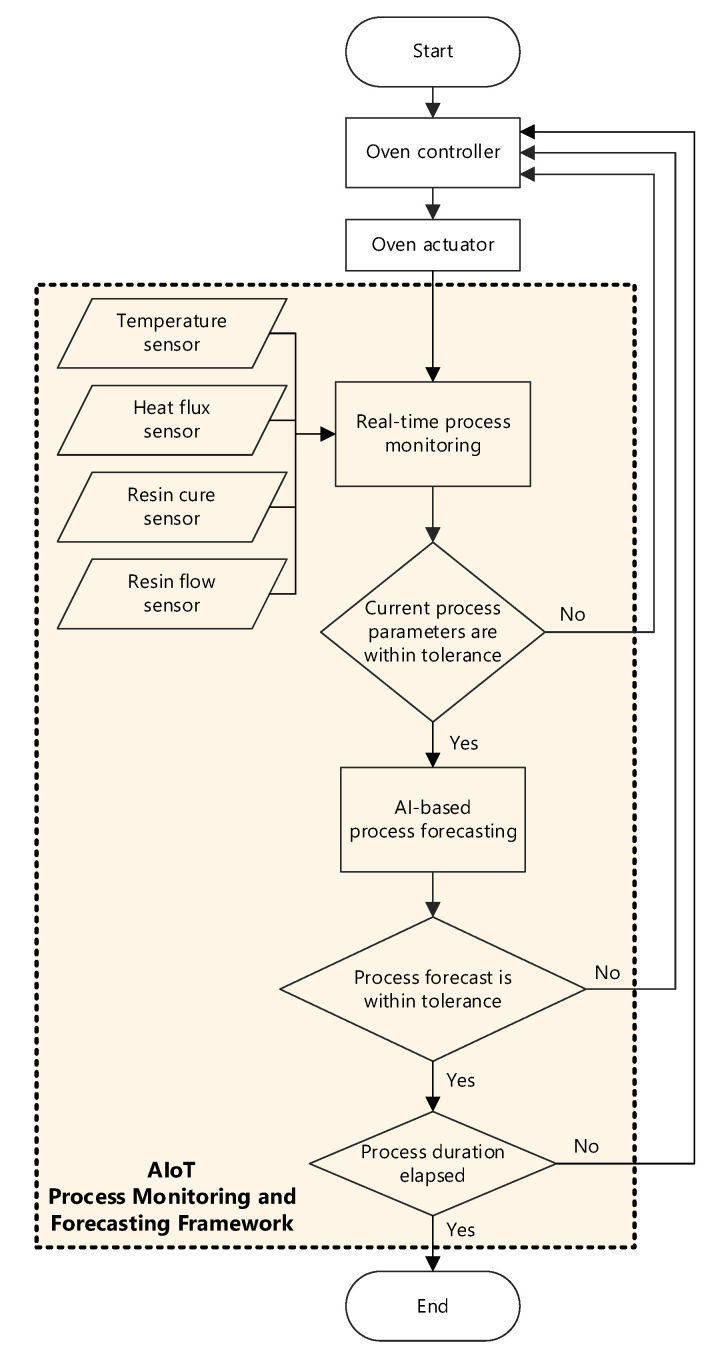
Process flowchart of the developed AIoT framework for composites manufacturing.

Depicted in a flowchart shown in Figure 5, the smart composites manufacturing process operates sequentially, initiating with process monitoring via sensors during the RI process in tandem with the curing oven controller. The acquired data are subsequently transferred to the server and stored on the IoT platform. On the server, real-time process data are visualised to facilitate monitoring. Simultaneously, a provided AI model analyses both the stored historical and real-time process data to forecast future process progressions. It is worth highlighting that the resolution and parameters investigated here are application-specific.

### 3.1. Resin Cure Monitoring and Visualisation

**Figure 6:**
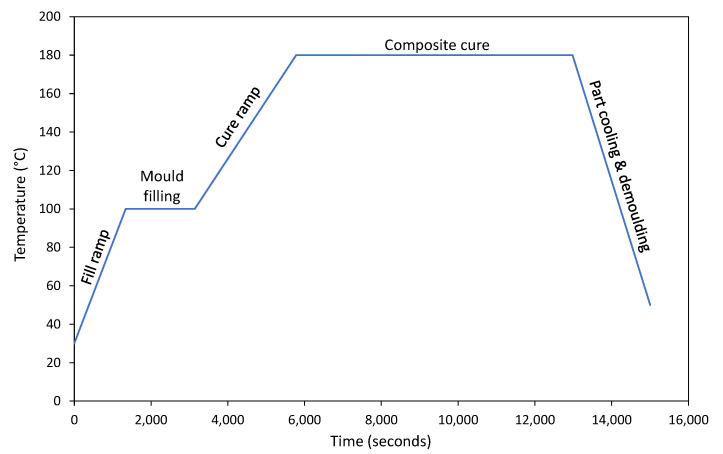
The resin manufacturer (Hexcel)-recommended RI and cure thermal profile.

During the RI process, the entire infusion layup, along with the subsequently injected resin, is heated in the oven. The resin utilised for infusion in this study is the HexFlow RTM6 [42], an aerospace-grade epoxy resin manufactured by Hexcel Corporation. The study adhered to the manufacturer-recommended infusion and curing thermal profile [42], as depicted in Figure 6. The process commenced with the fill ramp, during which the resin was gradually heated to 100 °C to lower its viscosity and facilitate resin permeation through the preform. Subsequently, the temperature was maintained constant during the ensuing resin injection stage to ensure thorough resin impregnation at a consistent viscosity level. Following the completion of resin injection, the entire composite underwent heating to 180 °C to trigger the epoxy resin curing process. Consistent with the manufacturer’s guidance, the product was cured at 180 °C for a duration of 2 h to achieve its optimal degree of cure and glass transition temperature. Upon completion of curing, the composite product was cooled to facilitate safe demoulding.

To monitor composite curing progression, the resin’s dielectric properties were closely observed [33,35]. Curing transforms liquid resin into a solid, three-dimensional polymer matrix via a cross-linking reaction, altering its dielectric properties [34,36]. These changes correlate with the resin’s Tg, obtainable through thermal and mechanical analyses like differential scanning calorimetry (DSC) and dynamic mechanical analysis (DMA). As the resin’s Tg is directly proportional to the composite’s degree of cure and resultant mechanical properties, exploiting this dielectric–Tg correlation enables effective cure monitoring. However, maintaining close and consistent sensor–resin contact is crucial for precision. In this study, the resin cure monitoring sensors manufactured by Synthesites [29] were utilised (Figure 4). In addition to resin cure progression, the composite temperature also needs to be monitored during curing, as the HexFlow RTM6 resin exhibits strong exothermic cure behaviour [35,36,42]. If left unmonitored, non-intentional deviations in the cure thermal profile may lead to exothermic runaway, where the exothermic curing accelerates uncontrollably from the heat generated itself, leading to a rapid and dangerous increase in temperature, release of hazardous substances, and potentially fire.

**Figure 7:**
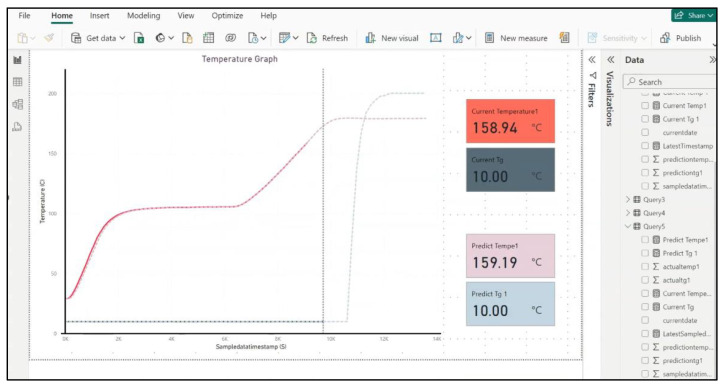
PowerBI interface of the proof-of-concept implementation showing real-time process visualisation and predictive process forecasting.

All sensors utilised were calibrated (using standardised methods such as the ice-point method) before implementation to ensure the reliability and accuracy of the measurements. For this proof-of-concept implementation, the real-time process visualisation and future process forecast are presented on the PowerBI interface, as shown in Figure 7. It is worth highlighting that the resolution of the AIoT framework is strongly dependent on the sampling rate of the process sensors.

### 3.2. AI-Based Resin Cure Forecasting

Recent advancements in IIoT have significantly increased the availability of large datasets from production lines. Such data collection is critical in the realisation of Industry 4.0 as it enables the development of process modelling and digital twinning, which in turn sprout the potential for process forecasting. However, production forecasting based on real-time acquired process data cannot be realised without computationally inexpensive process modelling approaches [7,12,14]. The approach we have taken in this work is to achieve process forecasting by developing and using a predictive AI model for this. This approach is favoured in the literature for similar applications [12,15,43]. AI-based process forecasting involves using AI models to make intelligent forecasts by identifying patterns in the sample or training dataset [1,9,44].

The appropriate selection of AI models for the process of interest is crucial, as different models yield varying performance, complexity (linear or non-linear), and target use cases [13]. In this proof-of-concept implementation, we combine long short-term memory (LSTM) and convolutional neural network (CNN) models to develop a hybrid process forecasting tool. This CNN-LSTM hybrid model [17,45] has proven effective in similar applications that analyse historic and present temperature data to forecast future thermal profiles [17,46,47,48]. More specifically, the hybrid CNN-LSTM model is recognised in the literature for its strong performance on datasets that exhibit non-linear relationships [12,48]. The CNN component of the CNN-LSTM model features a convolutional layer and a pooling layer. The rectified linear unit activation function was adopted in the convolutional layer, with the pooling layer tasked to narrow the features to create the input for the LSTM component. The LSTM model hosts a layer of 100 memory blocks that adopt the same activation function. Adam’s Stochastic Algorithm [49] was adopted to optimise the weights of the model with a learning rate of 0.001, with the model trained for 100 epochs.

A series of real-world RI experiments (setup shown in Figure 4) were conducted to collect the necessary process data for the development and training of the process forecasting tool. However, due to the high costs associated with data acquisition through experimental approaches [14,15], finite element modelling—developed from acquired process data and boundary conditions—was also utilised to generate supplemental data for training the AI-based forecasting tool. The generated dataset was randomly sampled and validated to be accurate (within acceptable tolerances) and reliable. Of the total training data acquired, 70% were used for training the forecasting model, while the remaining 30% were allocated for model testing and validation.

**Figure 8:**
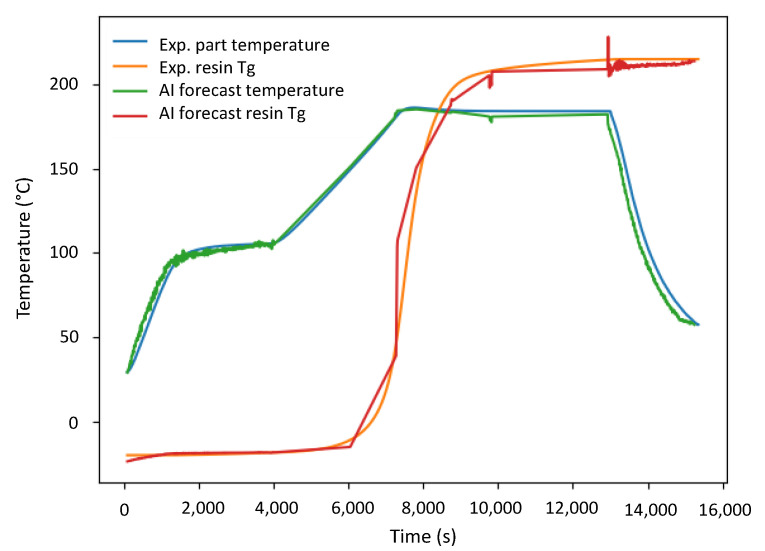
Comparison of the experimentally monitored parameters (Exp.) versus the AI forecasts (AI).

In this proof-of-concept implementation, the process forecasting error was assessed using the root mean square error (RMSE) to quantify the CNN-LSTM forecast accuracy. The process parameters monitored and to be forecasted are the part temperature (°C) and the resin’s Tg evolution (°C). Given that the prediction mechanism of the CNN-LSTM model incorporates a level of stochasticity, the average result from 10 forecast repetitions is depicted in Figure 8.

Following the manufacturer-recommended infusion and curing thermal profile shown in Figure 6, with the experimentally monitored part temperature ranging from 30 °C to 185 °C, the developed AI forecasting tool achieved an average RMSE of 5.02 °C. Conversely, with the resin’s Tg evolution ranging from −20 °C to 220 °C, the tool yielded an average RMSE of 8.0 °C. The results obtained demonstrate a satisfactory process forecasting capability, which can be further enhanced as more process data become available or are monitored. Due to the hazardous potential thermal runaway of the resin investigated, intentional process deviations were not introduced [42]. Nevertheless, in real-world employment, application-specific extensions to this proof-of-concept implementation can be easily incorporated. Working with known resin characteristics, the allowable process deviations alongside appropriate process corrective (or termination) actions can be designed accordingly to pre-empt fully developed defects or damage [13,20,50].

## 4. Summary and Conclusions

In this paper, an AIoT framework is introduced to streamline composites manufacturing operations via real-time process monitoring and forecasting. This is an IoT- and AI-driven composites manufacturing framework that comprises three main components: (i) an array of temperature, heat flux, dielectric, and flow sensors for process data acquisition, (ii) an IoT-based platform for instantaneous sensor data integration and visualisation, and (iii) an AI-based model for production process forecasting. It can provide valuable insights into the real-time progression of processes informed by sensors and utilise the acquired data to forecast future process advancements. The monitoring of the resin cure and temperature data streams creates a sufficiently accurate depiction of the process for real-time process monitoring and defect detection. Extensions towards modelling the multitudinous chemo-physical processes are also possible by the introduction of their relevant process governing equations (e.g., Darcy’s law for resin injection and Newton’s law of cooling for part heating/cooling).

A proof-of-concept implementation investigating a real-world composite manufacturing case study was developed to showcase the proposed AIoT framework. The case study investigated the resin injection and curing stages of the RI process, with a particular focus on the composite’s thermal profile and cure evolution across different stages of the process. The presented case study results demonstrated satisfactory outcomes, affirming the capabilities and suitability of the proposed smart composites manufacturing framework for relevant applications. The proposed AIoT framework holds great potential to propel the traditional composites manufacturing industry toward a digitalised manufacturing era at the Industry 4.0 level. Given the inherent uncertainties in composites manufacturing, the proposed framework serves as a valuable tool for facilitating cycle time optimisation, ensuring complete mould filling, identifying quality issues in real time, and preventing thermal runaway or degradation from the exothermic curing reaction.

Building upon this foundation, a potential future direction could be the automation of artificial intelligence-assisted active control mechanisms (e.g., corrective or preventive actions) when undesirable process conditions are detected or forecasted. Recall the two key challenges of contemporary composites manufacturing discussed earlier: material variabilities and process inconsistencies. The first step to the First Time Right approach would be developing appropriate statistical models to accurately model the undesirable process variabilities. Customised process optimisation strategies in response to different specific deviations or defects can then be investigated and implemented. Lastly, similar to another recent paper investigating the process monitoring and forecasting of the automated tape layering process [15], this paper focuses only on specific stages of the LCM process. In the future, by digitalising all the individual stages of LCM and integrating them, a robust IIoT or AIoT framework that encompasses all the cascading stages of LCM could be developed, achieving the final goal of Composites 4.0.

## Data Availability

Derived from an industry project, restrictions apply to the availability of these data. Data may be made available on request, on a case-by-case basis.
